# Emergency therapy of maternal and fetal arrhythmias during pregnancy

**DOI:** 10.4103/0974-2700.62116

**Published:** 2010

**Authors:** Hans-Joachim Trappe

**Affiliations:** Department of Cardiology and Angiology, University of Bochum, Germany

**Keywords:** Antiarrhythmic drugs, automatic implantable cardioverter defibrillator, cardiac arrhythmias, pregnancy, sudden death

## Abstract

Atrial premature beats are frequently diagnosed during pregnancy (PR); supraventricular tachycardia (SVT) (atrial tachycardia, AV-nodal reentrant tachycardia, circus movement tachycardia) is less frequently diagnosed. For acute therapy, electrical cardioversion with 50–100 J is indicated in all unstable patients (pts). In stable SVT, the initial therapy includes vagal maneuvers to terminate tachycardias. For short-term management, when vagal maneuvers fail, intravenous adenosine is the first choice drug and may safely terminate the arrhythmia. Ventricular premature beats are also frequently present during PR and benign in most of the pts; however, malignant ventricular tachyarrhythmias (sustained ventricular tachycardia [VT], ventricular flutter [VFlut] or ventricular fibrillation [VF]) may occur. Electrical cardioversion is necessary in all pts who are in hemodynamically unstable situation with life-threatening ventricular tachyarrhythmias. In hemodynamically stable pts, initial therapy with ajmaline, procainamide or lidocaine is indicated. In pts with syncopal VT, VF, VFlut or aborted sudden death, an implantable cardioverter-defibrillator is indicated. In pts with symptomatic bradycardia, a pacemaker can be implanted using echocardiography at any stage of PR. The treatment of the pregnant patient with cardiac arrhythmias requires important modifications of the standard practice of arrhythmia management. The goal of therapy is to protect the patient and fetus through delivery, after which chronic or definitive therapy can be administered.

## INTRODUCTION

Both supraventricular and ventricular tachyarrhythmias are not uncommon during pregnancy.[1,2] However, patients, relatives and physicians are frequently worried about ectopic beats and sustained arrhythmias.[3,4] One should question whether arrhythmias should be treated in the same way as outside pregnancy because all commonly used antiarrhythmic drugs cross the placenta.[5] Furthermore, the pharmacokinetics of drugs are altered in pregnancy and blood levels need to be checked to ensure maximum efficacy and avoid toxicity.[6–8] The major concern about antiarrhythmic drug therapy during pregnancy is the potential adverse effects on the fetus. In addition, in every pregnant woman with an arrhythmia, fetal cardiac assessment is necessary because fetal tachyarrhythmias can occur alone or combined with tachyarrhythmias of the mother.[9,10] For these reasons, treatment of cardiac arrhythmias in intensive care and emergency medicine is difficult during pregnancy. Correct therapy based on an understanding of the mechanism that caused the arrhythmia may not only be lifesaving for the mother but may also play an important role for the fetus.[11,12] The purpose of the present manuscript is to summarize new strategies for pregnant woman with supraventricular or ventricular tachyarrhythmias requiring emergency treatment.

## MATERNAL ARRHYTHMIAS DURING PREGNANCY

### Incidence and first manifestation

Supraventricular or ventricular tachyarrhythmias can become more frequent during pregnancy or they may develop for the first time.[13] An increase in the incidence of cardiac arrhythmias has been reported during pregnancy in patients with and without identifiable heart disease.[14] New onset or increased frequency of supraventricular or ventricular tachyarrhythmias was reported during pregnancy in patients with preexcitation syndromes or other causes.[15] Increased sympathetic activity during pregnancy has been proposed as a mechanism for increased incidence of arrhythmias.[1,16] Occurrence of cardiac tachyarrhythmias may also be related to physiological changes occurring during pregnancy, such as increased heart rate, decreased peripheral resistance and increased stroke volume.[17] Lee *et al*.[18] reported a low risk of first onset of paroxysmal supraventricular tachycardia (SVT) during pregnancy with an incidence of 4%. In the 107 patients with accessory pathway-mediated tachycardia, 7 patients had the first onset of tachycardia during pregnancy. In the 100 patients with atrioventricular-nodal reentrant tachycardia, one patient had the first onset of tachycardia during pregnancy. Ventricular tachycardia (VT) is rarely observed during pregnancy. Nakagawa *et al*.[19] studied 11 pregnant women who experienced new-onset ventricular arrhythmias during pregnancy. The onset of their first episode of ventricular arrhythmia was distributed equally over the three trimesters. The authors concluded that various hemodynamic and neurohumoral changes associated with pregnancy play an important role in ventricular arrhythmogenesis.[19] In women with well-known recurrent episodes of SVTs, 14 of 63 patients (22%) with tachycardia in the pregnant and nonpregnant periods had exacerbation of symptoms during pregnancy.[18] Similar observations have been reported by others.[20,21]

### Types of arrhythmias

Shotan *et al*.[14] assessed the relationship between symptoms and cardiac arrhythmias in 110 consecutive pregnant patients without evidence of heart disease referred for evaluation of palpitations (group G I), dizziness and syncope. These patients were compared with 52 consecutive pregnant patients referred for evaluation of symptomatic functional precordial murmur (group G II). Sinus bradycardia (heart rate <60/min) recorded during Holter monitoring (1% in G I, 2% in G II; *P* = ns) and sinus tachycardia (heart rate >100/min) (9% in G I, 10% in G II; *P* = ns) were relatively rare, whereas there was a high frequency of sinus arrhythmias in both groups (61% in G I, 69% in G II; *P* = ns). Isolated atrial premature beats (APB) were seen in 56% in G I and 58% in G II (*P* = ns), complex APBs (5% GI and 0% G II patients, *P* = ns) or SVT (1% in G I, 6% in G II, *P* = ns) were observed rarely. Isolated ventricular premature beats (PVC) were recorded in 49% G I and 40% G II patients (*P* = ns), whereas the incidence of multifocal PVCs was higher in G I (12%) than in G II (2%) (*P* < 0.05). Ventricular tachycardia (VT) or ventricular fibrillation (VF) were not recorded in any of the patients.[14]

### Diagnostic procedures

Before initiating therapy, it is important to correctly diagnose the type and mechanism of the underlying arrhythmia so that the proper therapeutic modalities can be implemented. The pregnant patient with arrhythmias usually seeks medical attention because of palpitations, light-headedness, shortness of breath or anxiety. Clues for correct diagnosis and treatment come from findings during physical examination and correct analysis of the electrocardiogram (ECG). Knowing the ECG features of the different types of narrow (QRS width <0.12 s) or wide (QRS width >0.12 s) tachycardias are of extreme importance to obtain ECG documentation of the arrhythmia so that the pregnant woman can receive the correct treatment.

## FETAL ARRHYTHMIAS DURING PREGNANCY

### Incidence

Intrauterine, all types of arrhythmias can occur. They are frequently intermittent and may disappear until delivery or the neonatal period.[22,23] Fetal arrhythmias can carry a significant risk of morbidity and mortality; especially, when arrhythmias cause hydrops fetalis, where it is associated with fetal death or neurological damage.[24,25] In 2003, in the Swiss prospective FETCH-study, there was an 11% incidence of arrhythmias in 433 fetal echocardiographic examinations (http://www.neonat.ch). Among these arrhythmias, supraventricular premature beats were present in 79%, atrial fibrillation (AF) in 2%, SVT in 15% and atrio-ventricular blocks in the remaining 4%. It has been reported that AV-nodal reentrant tachycardia, ectopic atrial tachycardia or atrial flutter (AFlut) are serious and threatening rhythm disorders in the human fetus.[26] A fetal tachycardia of a moderate high rate with 1 : 1 retrograde conduction and poor cardiac tolerance can be due to a junctional ectopic tachycardia (JET).[27] In contrast to arrhythmias with a heart rate >100/min, high-degree AV-block with persistent fetal bradycardia can occur either in normal hearts or those with structural diseases.[28,29] There is a poor prognosis when high-degree AV block is associated with congenital heart disease. In some cases, the fetal congenital AV-block is caused by QT-prolongation or immune-diseases mediated.[30]

### Diagnostic procedures

The description of intrauterine AFlut by Carr and McLure in 1931 is probably the first published report. Blumenthal *et al.* documented intrauterine arrhythmias with the use of fetal electrocardiography in 1968. Currently, fetal echocardiography is the best method and remains the cornerstone for in utero diagnosis of arrhythmias.[31] It has been shown that the electrophysiologic mechanisms of fetal supraventricular tachyarrhythmias can be clarified with superior vena cava/aorta Doppler flow recordings.[32] For differentiation of supraventricular from ventricular arrhythmia cross-sectional echocardiography, M-mode and echo-Doppler have been used. Conventional fetal echocardiographic views of the heart were obtained to exclude structural heart malformation. It is possible to determine the atrial rate using M-mode echocardiography, while the ventricular rate is determined with the use of M-mode and/or echo-Doppler.

## ACUTE THERAPY OF SUPRAVENTRICULAR ARRHYTHMIAS IN THE PREGNANT WOMAN

### Narrow-QRS complex tachycardia

**Figure 1:**
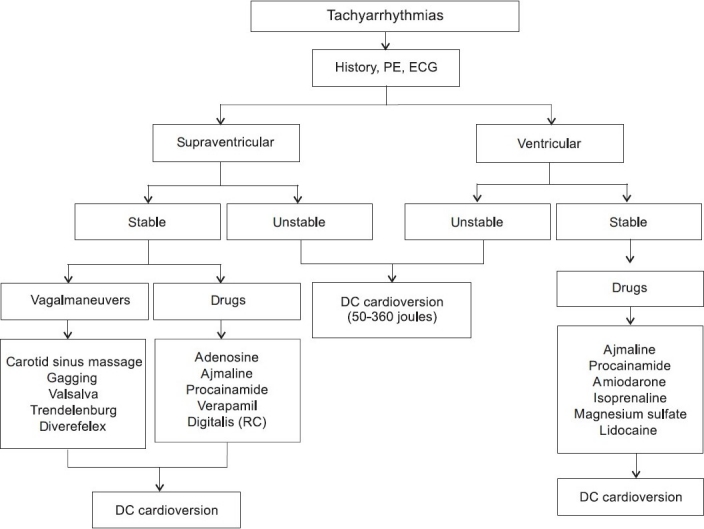
Treatment algorithm for pregnant patients with tachyarrhythmias. DC = direct current, ECG = electrocardiogram, PE = physical examination, RC = rate control

Narrow-QRS tachycardia is a cardiac rhythm with a rate faster than 100 beats/min and a QRS duration of <0.12 s.[34] The patient with narrow-QRS tachycardia usually seeks medical attention because of palpitations, light-headedness, shortness of breath or anxiety. In many patients with a narrow-QRS complex tachycardia, the tachycardia rate is very high (180-240 beats/ min) and therefore, after onset of the tachycardia the patient will arrive very soon thereafter in an intensive care unit for diagnosis and treatment. The definitive diagnosis of a narrow-QRS complex tachycardia can be made in most of the patients based on the 12-lead electrocardiography and clinical criteria. Acute treatment should be initiated based on the underlying mechanism. In regular narrow-QRS complex tachycardia (QRS width <0.12 s), vagal maneuvers should be initiated to terminate the arrhythmia or to modify AV conduction.[33,34] If this fails, adenosine or calcium channel blockers (verapamil) are the drugs of first choice [Figure 1]. Specific antiarrhythmic drugs should be avoided whenever possible in these conditions, because all commonly used antiarrhythmic drugs cross the placenta and may develop serious side effects to the fetus. The advantage of adenosine (9-18 mg iv as bolus) relative to intravenous calcium antagonists or beta-blockers relates to its rapidity of onset and short half-life.[35] In addition, the present report on human clinical experience with adenosine during pregnancy indicates no teratogenicity or other adverse effects to the fetus and it is as effective in terminating SVT (efficacy rates >90%) in pregnant women as it is in patients who are not pregnant. Longer acting agents (intravenous calcium channel blockers or cardioselective beta-blocking agents) are of limited value because of a possible increase of hypotensive and/or bradycardiac effects.[36] In patients with AV-nodal reentrant tachycardia, intravenous calcium channel blockers are acceptable drugs. The greatest experience has accrued with verapamil (10 mg iv over 3 min, 5 mg iv in woman with previous beta-blocker therapy and/or hypotension (RR_syst_ < 100 mmHg). Clinical studies of verapamil in pregnant women have not demonstrated adverse effects on either the patient or the fetus. However, intravenous administration of verapamil carries a risk of precipitating maternal hypotension and secondary hypoperfusion. In addition, verapamil is capable of causing fetal bradycardia, high-degree AV block and hypotension. Pregnancy is also related to an increased frequency of arrhythmias in previously asymptomatic patients with Wolff- Parkinson-White syndrome.[37] Therefore, ajmaline (50–100 mg iv over 5 min) is an alternative antiarrhythmic drug in emergencies, particularly in patients with accessory pathways; this has been well known for many years in nonpregnant patients with circus movement tachycardia.[38] There are no sufficient data regarding teratogenicity or other adverse effects to the fetus when ajmaline is used. Therefore, ajmaline should be avoided during the first trimester and only used when other therapeutic alternatives are not present or even unsuccessful. If vagal maneuvers and/or unspecific or specific drugs are ineffective to terminate SVT, direct current (DC) cardioversion (10-50 J) is well tolerated and effective to terminate the arrhythmia.[39] In a very few pregnant patients with otherwise untreatable tachycardia, either by drugs or by DC current energy, a ‘rescue’ radiofrequency ablation is indicated and possible with excellent results and shows no serious side effects for the pregnant woman or the fetus.[40]

### Atrial fibrillation and atrial flutter

Any arrhythmia can occur in the pregnant woman, and the frequency and symptomatic severity of arrhythmias may be increased during pregnancy. Although atrial fibrillation (AF) and atrial flutter (AFlut) are very frequent arrhythmias in adult non- pregnant patients, AF and AFlut are unusual in the absence of structural heart disease.[41] Obviously, hemodynamic, hormonal, autonomic and emotional changes related to pregnancy may contribute. In the event of hemodynamic embarrassment caused by AF/AFlut with rapid ventricular response, electrical DC cardioversion is usually successful with 50–100 J.[42] Cardioversion should always be performed in a synchronized mode. In AF/ AFlut with well-tolerated hemodynamics, quinidine has the longest record of safety in pregnant women for chemical cardioversion; however, other class Ia/Ic antiarrhythmia drugs are also safe for short-term use.[10] Rate-slowing drugs (β-blocking agents) should be administered before starting quinidine because of its vagolytic effect on the atrioventricular (AV) node. Rate control of AF is possible using digoxin, β-blocking agents and/or verapamil. However, intravenous administration of verapamil carries a risk of precipitating maternal hypotension and secondary hypoperfusion causing fetal bradycardia, high-degree AV block and hypotension.

### Atrial premature beats

Atrial premature beats (APB) in pregnant woman with structurally normal hearts are benign.[10] Atrial premature beats become more frequent during pregnancy or may develop for the first time and many patients are worried about it.[43] Patient education and reassurance are the first level of intervention of this benign condition. Exacerbating factors, such as chemical stimulants should be identified and eliminated. Drug therapy is not needed in the vast majority of pregnant women. In patients who remain highly symptomatic, treatment with selective β-adrenergic receptor-blocking agents should be considered. The few randomized studies of their use in pregnancy have yielded conflicting results regarding their effectiveness and safety. β-Blocking agents readily cross the placenta and could, in large doses, cause a relative fetal bradycardia. Preferred drug for treatment of APBs is a β1-selective agent (metoprolol). In contrast, β2-blocking agents are associated in some cases with reduced utero-placental perfusion and/or fetal growth retardation and should not be given.[44]

## ACUTE THERAPY OF VENTRICULAR ARRHYTHMIAS IN THE PREGNANT WOMAN

One of the most important problems in intensive care, emergency medicine and cardiac rhythmology are to take care of pregnant patients with recurrent VT, ventricular flutter or VF. Management of cardiac arrest due to life-threatening ventricular tachyarrhythmias is essential to prevent sudden cardiac death in the mother and the fetus. However, treatment of the underlying arrhythmia requires a correct diagnosis. This is possible in the majority of patients using a 12-lead surface electrocardiogram.

### Wide-QRS complex tachycardia

Since a drug given for the treatment of SVT may be deleterious to a patient with VT, the differential diagnosis of a broad QRS tachycardia is critical. Wide-QRS complex tachycardias (QRS duration >0.12 s) often pose a difficult diagnostic and therapeutic problem.[34] Errors are made because emergency care professionals wrongly consider VT unlikely if the patient is young and hemodynamically stable, and they are often unaware of the ECG findings that quickly and accurately distinguish VT in more than 90% of cases. To make the right diagnosis, it is ideal to have a 12-lead ECG. Diagnostic clues for differentiation of VT from SVT are findings in lead V1 and V6; in addition, a QRS of 0.14 s or more favors a diagnosis of VT. There are several possible mechanisms of wide-QRS complex tachycardia.

**Figure 2:**
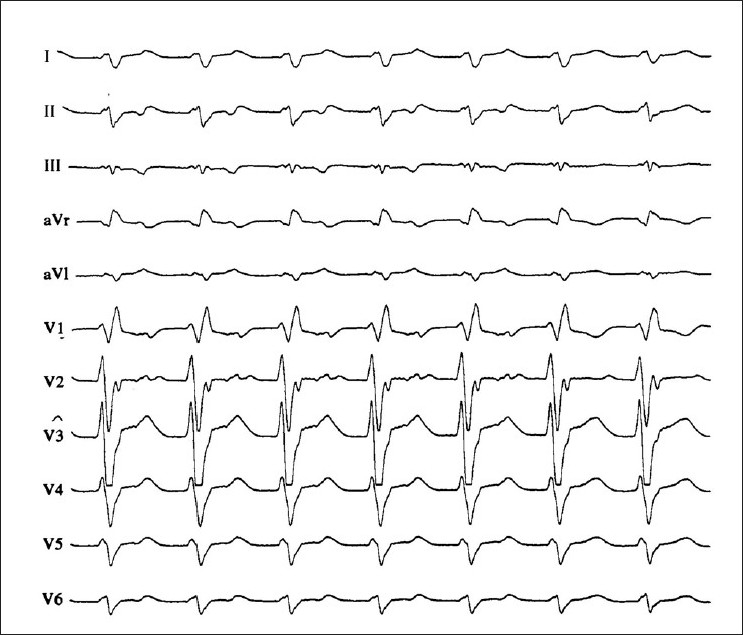
A 12-lead-ECG of a patient with wide-QRS complex tachycardia from a 28-year-old pregnant woman (31 week of gestation). The tracing shows a right bundle branch block like morphology with a triphasic appearance in V1, but an R/S ratio of less than 1 in V6 and a northwest QRS axis, typical for a ventricular tachycardia. There is one-to-one ventriculoatrial conduction during the tachycardia. The p waves are negative in leads II and III

Although sustained (duration >30 s) VT is rare in pregnant women, there are some reports that VT (when occurring) originates in the patient with a normal heart mainly from the right ventricular outflow tract.[34] Idiopathic left VT also occurs in pregnant patients with structurally normal hearts. In contrast to pregnant patients with normal left ventricular function, there is a poor prognosis when VT is associated with structural heart disease.[10] For acute treatment differentiation of VT – hemodynamically unstable or stable – is essential. If at any time VT is becoming unstable or if there is evidence of fetal compromise, DC countershock (50–100 J) should be delivered immediately [Figure 1]. If a DC shock of 50-100 J is unsuccessful, higher energy is mandatory (100–360 J) and without any risk for mother and children. ‘Conservative’ therapy is indicated in any patient with sustained VT and stable hemodynamics [Figure 2]. Acute therapy should start with intravenous procainamide or with ajmaline (50–100 mg iv over 5 min). Procainamide appears to be equally safe, is well tolerated and has not been associated with teratotoxicity, whereas the potential risk of ajmaline during pregnancy is unclear and administration should be limited for emergencies.[10] Another potential antiarrhythmic drug is lidocaine, which is not known to be teratogenic. Although several studies have shown some adverse effects (increase in myometrial tone, decrease in placental blood flow, fetal bradycardia), its use during the early months of pregnancy is not associated with a significant increase in the incidence of fetal defects.[39] Class III antiarrhythmic agents (such as sotalol, amiodarone) are very effective drugs in patients with ventricular tachyarrhythmias. During pregnancy, both drugs are of limited value: sotalol appears to be relatively safe, although there is a 3–5% risk of developing polymorphic or torsade de pointes tachycardia [[Fig F0003]]. In addition, the β-adrenergic properties of sotalol must be considered. Amiodarone is well known for its many and serious side effects both for the mother and the fetus, including hypothyroidism, growth retardation and premature delivery.[45,46] There is limited experience of amiodarone usage during pregnancy and thus treatment with this drug should be reserved for life-threatening conditions.[47] Magnesium is another drug with antiarrhythmic properties, particularly in patients with torsade de pointes tachycardia due to QT prolongation. It is known for a long time that in emergency circumstances, magnesium sulfate (dosage 1 to 2 g iv) is effective to treat and to suppress life-threatening ventricular tachyarrhythmias and should be administered over 1 to 2 min. Although this drug is associated with few side effects, maternal hypothermia and fetal bradyarrhythmias have been observed.[48] In a few cases, verapamil is effective in pregnant women with right/left ventricular outflow tachycardia.[49]

**Figure 3 F0003:**
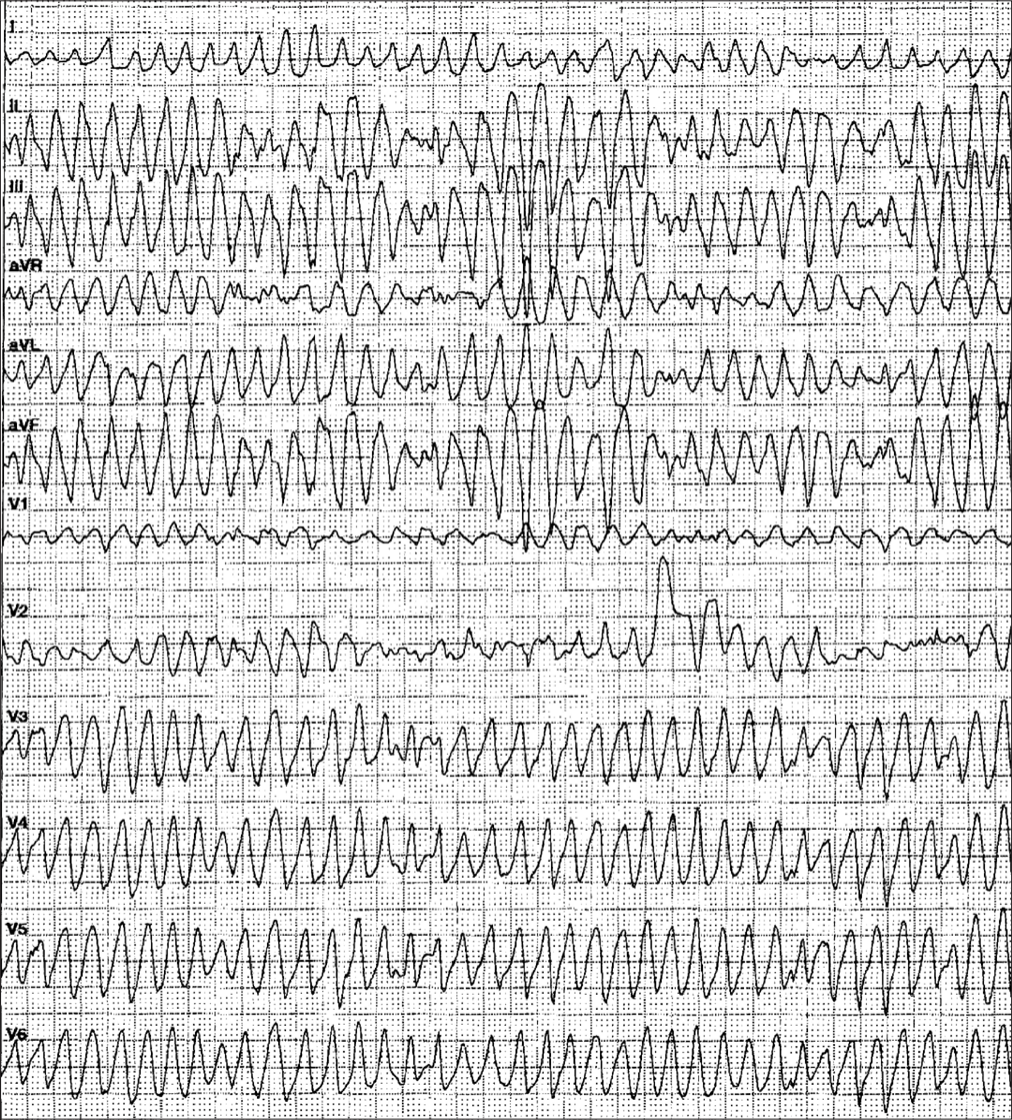
12-lead- ECG of a 34-year-old pregnant woman with a fast polymorphic ventricular tachycardia (25 week of gestation)

### Ventricular fibrillation and ventricular flutter

Life-threatening ventricular fibrillation (VF) or ventricular flutter (VFlut) can occur in any stage of pregnancy and is associated with a high risk of sudden cardiac death. In patients with VF or VFlut, DC defibrillation is the method of choice (100-360 J). Prompt cardiopulmonary resuscitation and early defibrillation by either DC-countershock or an automated external defibrillator (AED) significantly improve the likelihood of successful resuscitation from VF.[50] For long-term therapy, the implantable cardioverter-defibrillator is an excellent approach to terminate ventricular tachyarrhythmias and to prevent sudden death. There are a few reports on ICD therapy during pregnancy, and these studies clearly show that ICD implantation did not negatively influence pregnancy, delivery or fetal health.[51]

### Ventricular premature beats

Ventricular premature beats (VPB) in pregnant woman with structurally normal hearts are benign and therapy is usually not necessary.[10] Patient education and reassurance are the first level of interventions of this benign condition. Exacerbating factors, such as chemical stimulants should be identified and eliminated. In patients who remain highly symptomatic after all steps have been taken, treatment with selective β-adrenergic receptor-blocking agents is indicated. The few randomized studies of their use in pregnancy have yielded conflicting results regarding their effectiveness and safety. β-Blocking agents readily cross the placenta and could, in large doses, cause a relative fetal bradycardia. Preferred drugs for treatment of VPBs are β1- selective agents like metoprolol. In contrast, β2-blocking agents has been associated in some cases with reduced uteroplacental perfusion and/or fetal growth retardation, and hence should not be chosen for treating VPBs.[44] There is no indication for treatment with class III antiarrhythmic drugs due to their side effects and risk for proarrhythmia.[14]

## ACUTE THERAPY OF FETAL ARRHYTHMIAS

Management of fetal arrhythmias is very difficult and requires cooperation of different consultants (obstetrics, cardiology and neonatology). The problem with fetal tachyarrhythmias is the risk of hydrops fetalis and subsequent death.[52] Supraventricular tachycardias are the most common fetal tachycardias, whereas other arrhythmias are observed less frequently. An analysis of 11 studies reported from 1991 to 2002 showed a fetal SVT as the underlying arrhythmia in 73.2% and AFlut in 26.2%.[53] The incidence of hydrops fetalis was similar in those with AFlut or SVT (38.6 versus 40.5%) (*P* = ns). Intrauterine death was 8.0% in fetal AFlut and 8.9% in fetal SVT (*P* = ns).

### Maternal therapy

The treatment of fetal arrhythmias is possible by treating the mother or by treating the fetus directly. Antiarrhythmic agents that have been used to treat fetal arrhythmias include digoxin, β-blocking agents, verapamil, procainamide and quinidine. In addition, in cases of fetal ventricular tachyarrhythmias class I and III antiarrhythmic agents have been advocated.[6,13] Recently, Anderer *et al*. reported a 25-year-old pregnant woman with persistent fetal tachycardia (rate 267 beats/min) and subsequent hydrops fetalis.[52] The woman was treated with flecainide and digoxin, and the tachycardia converted to sinus rhythm. A few days later, no signs of fetal heart failure were present. In another publication, Khosithseth *et al.* described 3 cases with hydrops fetalis due to supraventricular tachyarrhythmias, successfully treated with amiodarone and digoxin or the combination of digoxin, procainamide and propranolol.[53]

### Direct fetal therapy

maternal therapy fails to suppress or sufficiently decreases the rate of fetal tachyarrhythmias, direct drug administration to the fetus is mandatory. Direct fetal treatment regimes have been used that consists of intraperitoneal and/or umbilical intravenous administration of different drugs. In addition, umbilical drug administration allows not only direct treatment but also drug monitoring. Hansmann *et al.* described 60 cases with fetal arrhythmias, of which 26 cases (43%) were with hydrops fetalis and 34 cases (57%) without. When tachyarrhythmias were refractory to transplacental treatment, fetal therapy was performed with direct umbilical drug administration.[54] Of those 60 cases, 54 were SVTs and 6 cases of AFlut. During the 9 years of the study, different drug regimes had been used. Twenty fetuses (77%) with tachyarrhythmias and hydrops fetalis survived, and all 34 nonhydropic fetuses survived. Therefore, direct fetal therapy is highly effective in SVT and AFlut and will lead to fetal survival. Amiodarone seems to be the drug of choice for direct therapy; however, there are also other effective drugs (digoxin, β-blocking agents, flecainide, adenosine).[55,56] Despite many side effects of amiodarone, the majority of children in the perinatal age are completely normal despite intrauterine therapy with amiodarone for tachyarrhythmias.

## CLINICAL IMPLICATIONS

During pregnancy, an increased incidence of maternal cardiac arrhythmias is observed, ranging from clinically irrelevant isolated atrial or ventricular premature beats to debilitating SVT and VT or VF. In all pregnant patients with tachyarrhythmias, evaluation of the underlying etiology and the degree of left ventricular function (dysfunction) is essential. Correct treatment of arrhythmias in the intensive care patient should be based on understanding the causal mechanism. In pregnant women with maternal and/or fetal arrhythmias, therapeutic strategies should be based on interdisciplinary cooperation (obstetrics, cardiology and neonatology). In general, acute therapy of arrhythmias during pregnancy is similar to that in the nonpregnant patient. However, special consideration should be given to potential teratogenic and hemodynamic adverse effects on the fetus. With this in mind, a successful pregnancy, for both mother and the fetus, can usually be the result.

## References

[1] Barron WM, Mujais SK, Zinamam M, Bravo EL, Lindheimer MD (1986). Plasma catecholamine responses to physiologic stimuli in normal human pregnancy. Am J Obstet Gynecol.

[2] Tawam M, Levine J, Mendelson M, Goldberger J, Dyer A, Kadish A (1993). Effect of pregnancy on paroxysmal supraventricular tachycardia. Am J Cardiol.

[3] Facchini M, Bauersfeld U, Fasnacht M, Candinas R (2000). Mütterliche Herzrhythmusstörungen während der Schwangerschaft. Schweiz Med Wochenschr.

[4] Page RL (1995). Treatment of arrhythmias during pregnancy. Am Heart J.

[5] Oakley C, Child A, Iung B, Task Force Members (2003). Expert consensus document on management of cardiovascular diseases during pregnancy. Eur Heart J.

[6] Joglar JA, Page RI (1999). Treatment of cardiac arrhythmias during pregnancy; safety considerations. Drug Saf.

[7] Rotmensch HH, Elkayam U, Frishman W (1983). Antiarrhythmic drug therapy during pregnancy. Ann Intern Med.

[8] Nakagawa M, Katou S, Ichinose M, Nobe S, Yonemochi H, Miyakawa I (2004). Characteristics of new-onset ventricular arrhythmias in pregnancy. J Electrocardiol.

[9] Allan L, Long WA (1990). Fetal arrhythmias. Fetal and neonatal cardiology.

[10] Chow T, Galvin J, McGovern B (1998). Antiarrhythmic drug therapy in pregnancy and lactation. Am J Cardiol.

[11] Copel JA, Kleiman CS (1989). Fetal echocardiography in the diagnosis and management of fetal heart disease. Clin Diagn Ultrasound.

[12] Cox JL, Gardner MJ (1993). Treatment of cardiac arrhythmias during pregnancy. Prog Cardiovasc Dis.

[13] Tan HL, Lie KI (2001). Treatment of tachyarrhythmias during pregnancy and lactation. Eur Heart J.

[14] Shotan A, Ostrzega E, Mehra A, Johnson JV, Elkayam U (1997). Incidence of arrhythmias in normal pregnancy and relation to palpitations, dizziness, and syncope. Am J Cardiol.

[15] Widerhorn J, Widerhorn AL, Rahimtoola SH, Elkayam U (1992). WPW syndrome during pregnancy: increased incidence of supraventricular arrhythmias. Am Heart J.

[16] Speranza G, Verlato G, Albiero A (1998). Autonomic changes during pregnancy: assessment by spectral heart rate variability analysis. J Electro Cardiol.

[17] Hunter S, Robson SC (1992). Adaptation of maternal heart in pregnancy. Br Heart J.

[18] Lee SH, Chen SA, Wu TJ, Chiang CE, Cheng CC, Tai CT (1995). Effects of pregnancy on first onset and symptoms of paroxysmal supraventricular tachycardia. Am J Cardiol.

[19] Nakagawa M, Katou S, Ichinose M, Nobe S, Yonemochi H, Miyakawa I, Saikawa T (2004). Characteristics of new-onset ventricular arrhythmias in pregnancy. J Electrocardiol.

[20] Bravermann AC, Bromley BS, Rutherford JD (1991). New onset ventricular tachycardia during pregnancy. Int J Cardiol.

[21] Navarro V, Nathan PE, Rosero H, Sacchi TJ (1993). Accelerated idioventricular rhythm in pregnancy: a case report. Angiology.

[22] Crosson JE, Scheel JN (1996). Fetal arrhythmias: diagnosis, and current recommendations for therapy. Progress in Pediatric Cardiology.

[23] Jaeggi E, Fouron JC, Drblik SP (1998). Fetal atrial flutter: diagnosis, clinical features, treatment, and outcome. J Pediatr.

[24] Lisowski LA, Verheijen PM, Benatar AA, Soyeur DJ, Stoutenbeek P, Brenner JI (2000). Atrial flutter in the perinatal age group: diagnosis, management and outcome. J Am Coll Vardiol.

[25] Strasburger JF, Cuneo BF, Michon MM, Gotteiner NL, Deal BJ, McGregor SN (2004). Amiodarone therapy for drug-refractory fetal tachycardia. Circulation.

[26] Fasnacht MS, Günthard J (2004). Fetale Kardiologie beinhaltet nicht nur fetale Echokardiographie. Pediatrica.

[27] Lupoglazoff JM, Dejoy I, Luton D, Magnier S, Azancot A (1999). Prenatal diagnosis of a familial form of junctional ectopic tachycardia. Prenat Diagn.

[28] Balmer C, Fasnacht MS, Rahn M, Molinari L, Bauersfeld U (2002). Long-term follow-up of children with complete atrioventricular block and impact of pacemaker therapy. Europace.

[29] Machado MV, Tynan M, Curry PV, Allen LD (1988). Fetal complete heart block. Br Heart J.

[30] Julkunen H, Kaaja R, Siren MK, Mack C, McCready S, Holthofer H (1998). Immune-mediated congenital heart block (CHB): identifying and counselling patients at risk for having children with CHB. Semin Arthritis Theum.

[31] Trappe HJ, Tchirikov M (2008). Herzrhythmusstörungen bei der Schwangeren und beim Fetus. Internist.

[32] Fouron JC, Fournier A, Proulx F, Lamarche J, Bigras JL, Boutin C (2003). Management of fetal tachyarrhythmia based on superior vena cava/aorta Doppler flow recordings. Heart.

[33] Sermer M, Colman J, Siu S (2003). Pregnancy complicated by heart disease: a review of Canadian experience. J Obstet Gynaecol.

[34] Wellens HJ, Conover MB (2006). The ECG in emergency decision making. 2nd ed.

[35] Wolbrette D (2003). Treatment of arrhythmias during pregnancy. Curr Womens Health Rep.

[36] Chow T, Galvin J, McGowern B (1998). Antiarrhythmic drug therapy in pregnancy and lactation. Am J Cardiol ;:I.

[37] Mozo de Rosales F, Moreno J, Bodegas A, Melchor JC, Fernandez Lebrez L, Aranguren G (1994). Conversion of atrial fibrillation with ajmaline in a pregnant woman with Wolff-Parkinson-White syndrome. Eur J Obsterics.

[38] Wellens HJ, Atie J, Penn OC, Gorgels AP, Brugada P, Smeets JL (1990). Diagnosis and treatment of patients with accessory pathways. Cardiol Clin.

[39] Page RL (1995). Treatment of arrhythmias during pregnancy. Am Heart J.

[40] Pagad SV, Barmade AB, Toal SC, Vora AM, Lokhandwala YY (2004). “Rescue” radiofrequency ablation for atrial tachycardia presenting as cardiomyopathy in pregnancy. Indian Heart J.

[41] Oakley C, Child A, Iung B, Task Force Members (2003). Expert consensus document on management of cardiovascular diseases during pregnancy. Eur Heart J.

[42] Walsh KA, Erzi MD, Denes P (1988). Emergency treatment of tachyarrhythmias. Med Clin North Am.

[43] Tan HL, Lie KI (2001). Treatment of tachyarrhythmias during pregnancy and lactation. Eur Heart J.

[44] Habib A, McCarthy JS (1977). Effects on the neonate of propranolol admininstered during pregnancy. J Pediatr.

[45] Khositseth A, Ramin KD, O'Leary PW, Porter CJ (2003). Role of amiodarone in the treatment of fetal supraventricular tachyarrhythmias and hydrops fetalis. Pediatr Cardiol.

[46] Vanbesien J, Casteels A, Bougatef A, De Catte L, Foulon W, DE Bock S (2001). Transient fetal hypothyroidism due to direct fetal administration of amiodarone for drug resistant fetal tachycardia. Am J Perinatol.

[47] Trappe HJ (2001). Amiodarone. Intensivmed.

[48] Cardosi RJ, Chez RA (1998). Magnesium sulfate, maternal hypothermia, and fetal bradycardia with loss of heart rate variability. Obstet Gynecol.

[49] Cleary-Goldmann J, Salva CR, Infeld JI, Robinson JN (2003). Verapamil-sensitive idiopathic left ventricular tachycardia in pregnancy. J Matern Fetal Neonatal Med.

[50] Trappe HJ (2005). Early defibrillation: where are we?. Dtsch Med WSchr.

[51] Natale A, Davidson T, Geiger MJ, Newby K (1997). Implantable cardioverter-defibrillators and pregnancy. A safe combination?. Circulation.

[52] Anderer G, Hellmeyer L, Tekesin I, Schmidt S (2005). Kombinationstherapie einer fetalen supraventrikulären Tachykardie mit Flecainid und Digoxin. Z Geburtshilfe Neonatol.

[53] Khositseth A, Ramin KD, O'Leary PW, Porter CJ (2003). Role of amiodarone in the treatment of fetal supraventricular tachyarrhythmias and hydrops fetalis. Pediatr Cardiol.

[54] Hansmann M, Gembruch U, Bald R, Manz M, Redel DA (1991). Fetal tachyarrhythmias: transplacental and direct treatment of the fetus – a report of 60 cases. Ultrasound Obstet Gynecol.

[55] Hubinont C, Debauche C, Bernard P, Sluysmans T (1998). Resolution of fetal tachycardia and hydrops by a single adenosine administration. Obstet Gynecol.

[56] Ishii K, Chiba Y, Sasaki Y, Kawamata K, Miyashita S (2003). Fetal atrial tachycardia diagnosed by magnetocardiography and direct fetal electrocardiography. A case report of treatment with propranolol hydrochloride. Fetal Diagn Ther.

